# Retrospective Comparison of Non-Skin-Sparing Mastectomy and Skin-Sparing Mastectomy with Immediate Breast Reconstruction

**DOI:** 10.1155/2011/876520

**Published:** 2011-08-14

**Authors:** Satoki Kinoshita, Kimihiro Nojima, Meisei Takeishi, Yoshimi Imawari, Shigeya Kyoda, Akio Hirano, Tadashi Akiba, Susumu Kobayashi, Hiroshi Takeyama, Ken Uchida, Toshiaki Morikawa

**Affiliations:** ^1^Department of Surgery, The Jikei University Kashiwa Hospital, 163-1 Kashiwashita, Kashiwa City, Chiba 277-8567, Japan; ^2^Department of Plastic-Surgery, The Jikei University Kashiwa Hospital, 163-1 Kashiwashita, Kashiwa City, Chiba 277-8567, Japan; ^3^Department of Breast and Endocrine Surery, The Jikei University School of Medicine, Tokyo 105-8461, Japan

## Abstract

*Background*. We compared Skin-sparing mastectomy (SSM) with immediate breast reconstruction and Non-skin-sparing mastectomy (NSSM), various types of incision in SSM. *Method*. Records of 202 consecutive breast cancer patients were reviewed retrospectively. Also in the SSM, three types of skin incision were used. Type A was a periareolar incision with a lateral extension, type B was a periareolar incision and axillary incision, and type C included straight incisions, a small elliptical incision (base line of nipple) within areolar complex and axillary incision. *Results*. Seventy-three SSMs and 129 NSSMs were performed. The mean follow-up was 30.0 (SSM) and 41.1 (NSSM) months. Respective values for the two groups were: mean age 47.0 and 57; seven-year cumulative local disease-free survival 92.1% and 95.2%; post operative skin necrosis 4.1% and 3.1%. In the SSM, average areolar diameter in type A & B was 35.4 mm, 43.0 mm in type C and postoperative nipple-areolar plasty was performed 61% in type A & B, 17% in type C, respectively. *Conclusion*. SSM for early breast cancer is associated with low morbidity and oncological safety that are as good as those of NSSM. Also in SSM, Type C is far superior as regards cost and cosmetic outcomes.

## 1. Introduction

The establishment of modern radical surgery for breast cancer started with standard radical mastectomy, conducted by William Stewart Halsted in 1882. This procedure consisted of extensive resection of overlying skin centered around the focus of cancer, the entire mammary gland, and the pectoralis major and minor muscles, as well as complete lymph node dissection. At a time when most cases of breast cancer were, what is now called, locally advanced or metastatic breast cancer, the procedure was considered an operative method to be implemented with curative intent because the three-year survival after surgery was over 40% and the outcomes of local control were astounding at the time [1]. 

The procedure was refined by his second-generation pupil, Cushman Davis Haagensen, profoundly influencing many surgeons around the world [2]. 

Halsted's procedure is based on the theory that breast cancer progresses anatomically, that is, from the mammary gland via the regional lymph nodes to the entire body. It was therefore essential to resect the regional lymph nodes and intervening lymphatics and to remove the entire mammary gland with radical surgery. This procedure became the basis for further extension of the surgical procedure, that is, internal mammary and supraclavicular lymph node dissection, after the long-term postoperative results peaked in the 1920–30s. This theory, however, rapidly lost favor after Fisher et al. introduced new concepts [3–5], and the usefulness of extended surgery for improving the prognosis was refuted by a clinical trial in the early 1980s [6]. On the other hand, the gradual movement toward limited surgery that started in the 1950s arose primarily because the detection of breast cancer at an early stage became possible, which was further reinforced by the requests for this surgery from women at the time. The transition to modified radical mastectomy progressed rapidly from 1975 to 1980 in the USA [7], and in Japan the procedure became mainstream in the late 1980s [8]. Later, after the NSABP (National Surgical Adjuvant Breast Project) protocol B-06 was conducted in 1985 [9], breast conserving surgery (BCS) was chosen more frequently, and the long-term (20-year) results demonstrated that BCS produced outcomes comparable to mastectomy [10, 11]. 

Today local control of breast cancer is the major objective of surgical treatment and considered a part of systemic therapy [12]. BCS is now the mainstream of breast cancer treatment. Even now, however, about one-third of women with breast cancer choose mastectomy, based on the size or site of the lesion and the presence of an extensive intraductal lesion [13]. 

Skin-sparing mastectomy (SSM) with immediate breast reconstruction reported by Toth and Lappert in 1991 is generally acknowledged to be the method that can achieve radical cure and resolve cosmetic issues [14]. At our hospital, we have adopted this method in cooperation with plastic surgeons and have produced excellent results since 2003. 

In the following sections, we report an overview of the findings and a retrospective case control study of skin-sparing mastectomy (SSM) and non skin-sparing mastectomy (NSSM) performed by a single surgeon during the same period. 

## 2. Patients and Methods

The subjects were 202 female Japanese patients who underwent mastectomy by a single surgeon (SK) at the Jikei University Kashiwa Hospital during the period from July 2003 to June 2010. Of these patients, 73 were assigned to the SSM group and 129 to the NSSM group. 

In the SSM group, removal of the nipple with/without areola complex, biopsy scars (excluding the core needle biopsy scar), and the entire breast parenchyma were planned [15]. Immediate breast reconstruction was performed by a plastic surgeon in all patients in the SSM group. 

**Figure 1:**
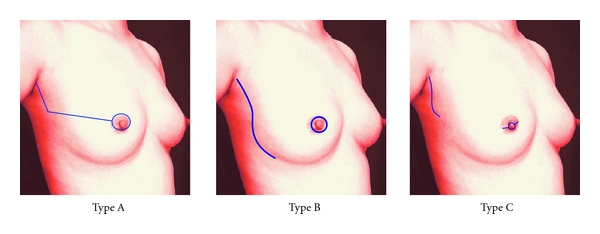
Classification of skin incisions for SSM.

In the SSM group, the patients were assigned to undergo three types of skin incision. Type A was a periareolar incision with a lateral extension (the so-called “tennis racquet”), type B was a periareolar incision and axillary incision, and type C included straight incisions, a small elliptical incision (base line of nipple) within areola complex (the so-called “areolar sparing”) and axillary incision (Figure 1). 

Appropriate adjuvant therapy was carried out for all patients based on their own choice after they underwent postoperative pathological examination and adequate informed consent was obtained. 

The chi-square test and *t-*test were used in the statistical analysis. The cumulative overall survival (OAS), cumulative distant disease-free survival (DDFS), and cumulative local disease-free survival (LDFS) were calculated by the Kaplan-Meier method, and a significant difference was evaluated by the Wilcoxon test (*P* ≤ 0.05). 

## 3. Results

Table 1 shows the patient (73 in the SSM group and 129 in the NSSM group) and tumor characteristics and tumor staging determined based on the American Joint Committee on Cancer Staging System. 

The mean age was 47.0 ± 9.0 (31–71) years in the SSM group and 57.7 ± 11.9 (31–83) years in the NSSM group, significantly lower in the former group (*P* < 0.000). The mean follow-up period in the SSM group was 30.0 ± 22.6 (1–85) months, which was significantly shorter than the 41.1 ± 21.3 (1–86) months in the NSSM group (*P* < 0.000). Stage 0 noninvasive cancer accounted for 15.1% of the SSM group and 7.8% of the NSSM group (*P* = 0.1). Neoadjuvant chemotherapy (NAC) was carried out in four cases in the SSM group (5.5%) and in five cases in the NSSM group (3.9%) (*P* = 0.6). 

Table 2 shows an overview of the operative procedures. In the SSM group, 48.0% of the patients underwent both total mastectomy (Bt) and axillary lymph node dissection (ALND), 34.2% underwent sentinel lymph node biopsy (SLNB) alone, and 17.8% additionally underwent ALND after SLNB. In the NSSM group, the percentages were 75.9%, 20.2%, and 3.9% respectively. The average time required for mastectomy was 140 minutes in the SSM group and 130 minutes in the NSSM group (*P* = 0.06); the intraoperative blood loss was 212 g in the SSM group and 197 g in the NSSM group (*P* = 0.5). 

**Figure 2:**
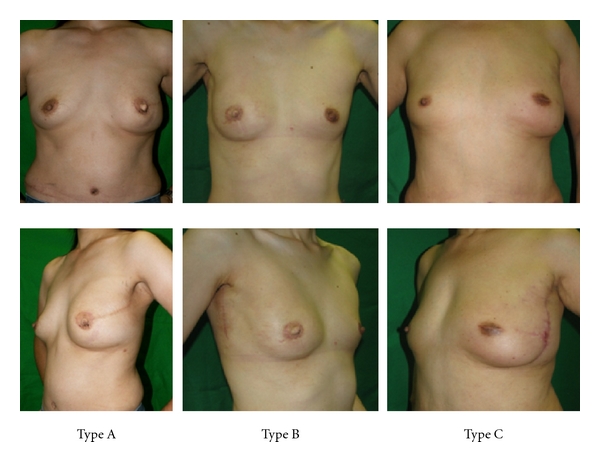
Appearance of the breast following SSM and reconstruction with a TRAM flap. Type A: left breast, type B: right breast, and type C: left breast.

Table 3 shows the type of skin incision and type of reconstruction, and Figure 2 shows the appearance of each type after reconstruction. Also in Table 3, the number of cases was compared between the first half of the study (July 2003 to June 2007) and the second half of the study (July 2007 to June 2010) in the SSM group alone—26 cases (35.6%) were in the first half and 47 cases (64.4%) were in the second half. The average operative duration decreased from 148 minutes to 132 minutes (*P* = 0.03), and the average intraoperative blood loss also decreased from 232 g to 196 g (*P* = 0.27). While type A accounted for 84.6% in the first half of the study, type B and C accounted for 53.2% and 38.3%, respectively, in the second half of the study (*P* < 0.000). The percentage of DIEP flap breast reconstructions increased from 15.4% in the first half to 53.2% in the second half (*P* = 0.002). The average length of the long axis of the periareolar incision was 3.7 cm in the SSM group. The average diameter was 35.4 mm in types A and B and 43.0 mm in type C, respectively (*P* = 0.0002). 

Table 4 shows the relation between nipple-areolar plasty and type of incision. Postoperative nipple-areolar plasty was requested in 48 cases (88.9%) and received in 33 cases (61.1%) of types A and B and in 3 cases (16.7%) of type C, respectively (*P* = 0.001). 

Table 5 shows a summary of postoperative complications. Skin necrosis that required debridement and further treatment was seen in 4.1% of the SSM group and in 3.1% of the NSSM group, showing no significant difference between the two groups (*P* = 0.69). In the SSM group, flap loss and deep vein thrombosis (DVT) due to circulatory insufficiency were each seen in 1.4%, fat lysis in the flap associated with infection that required surgical approaches and a hernia at the donor site each occurred in 2.7%. 

Table 6 shows the complications observed in smokers and nonsmokers in the SSM group. The combined incidences of skin and flap-related problems and DVT were 20% and 5.7%, respectively (*P* = 0.06). As regards local recurrence during the follow-up period, two episodes were reported in the SSM group (2.7%) and five episodes in the NSSM group (3.9%). One case of cancer death was reported in the SSM group (1.4%) and six cases were reported in the NSSM group (4.7%). 

**Figure 3:**
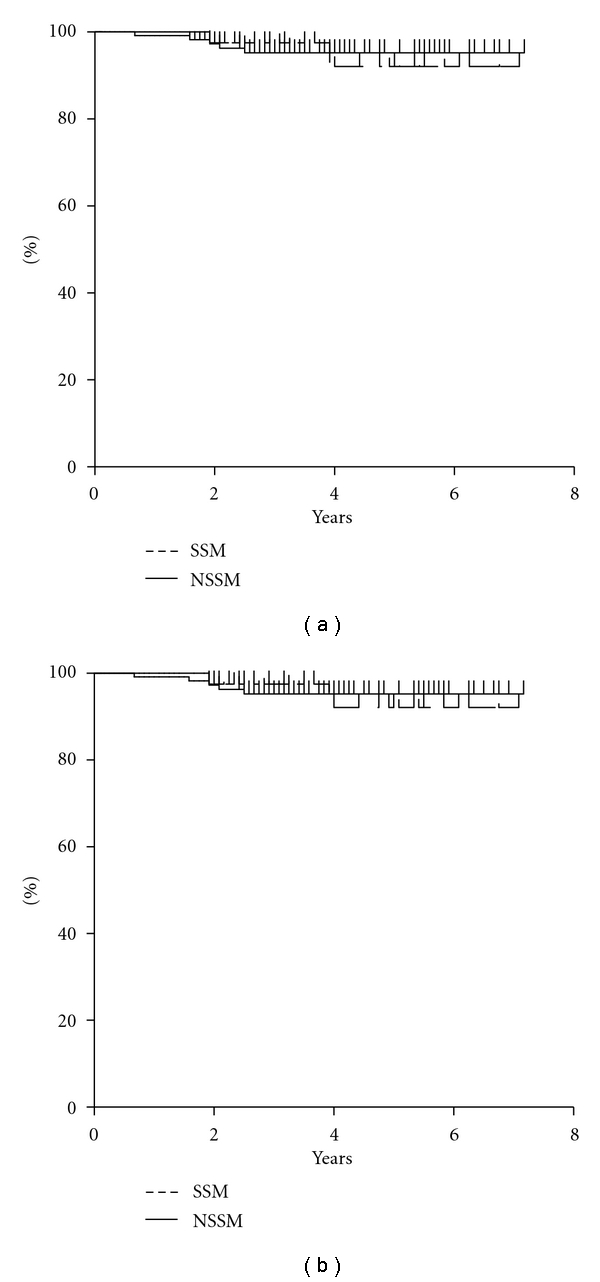
Kaplan-Meier survival curve for SSM and NSSM. (a) Local disease-free survival *P* = 0.75. (b) Overall survival *P *= 0.69.

Figure 3 shows the local disease-free survival (LDFS) and overall survival (OAS) determined by the Kaplan-Meier method. Seven-year LDFS was 92.1% in the SSM group and 95.2% in the NSSM group (*P* = 0.75), and seven-year OAS was 96.9% in the SSM group and 90.1% in the NSSM group (*P* = 0.69). There was no significant difference in rates between the two groups. 

## 4. Discussion

SSM with immediate breast reconstruction has rapidly spread during the past 20 years, and its origin dates back to subcutaneous mastectomy, first performed by Freeman in1962 [16].

In SSM, the nipple-areolar complex and all biopsy scars excluding the core needle biopsy scar are resected, inframammary fold and most of the native breast skin are preserved, and the entire breast parenchyma is removed. Usually SSM is followed by immediate breast reconstruction, through which better cosmetic outcomes are produced, the anesthetic risk and the patient's emotional trauma from the loss of a breast are reduced, and, ultimately, cost-effectiveness is achieved [17, 18]. 

The mean age of the SSM group is generally lower than that of the NSSM group [15]. This may reflect bias not only among patients who choose the operative procedure but also oncological surgeons who propose the operative procedure. 

We believe that the follow-up period was significantly shorter in the SSM group in this case series because 64% of SSMs were performed in the second half. 

In view of the anatomical course of ducts, resection of the nipple-areolar complex has been considered to be essential because the tumor cells may spread to the adjacent ducts. The involvement of tumor cells at the nipple-areolar complex is reported to occur in about 3–10%, except for the extremely high percentage of 58% reported in one study [13, 15]. On the other hand, Simmons et al. examined the nipple and areola separately and reported that areolar involvement was seen in just 0.9% [19]. At our institution, during the second half of this case series we tried an approach that uses the type C skin incision while taking account of the information obtained from preoperative, contrast-enhanced CT/MRI to achieve better cosmetic outcomes and obtained the positive outcomes seen in the study by Simmons et al. [20], although our study period was relatively short. 

**Figure 4:**
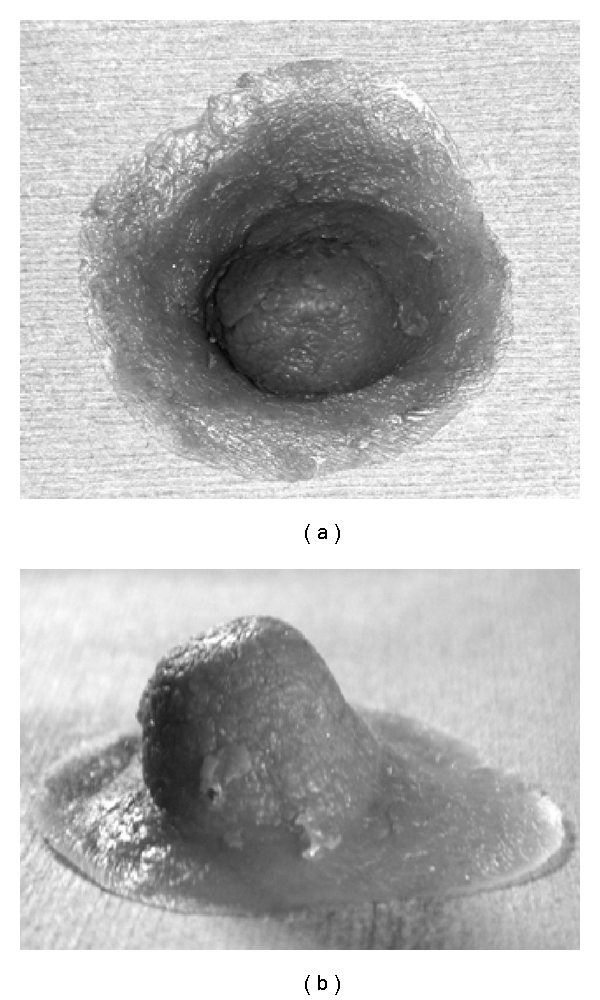
nipple-areolar complex made with silicon material.

The average areolar diameter of type C was significantly larger than that of types A and B. Therefore, we consider that areolar sparing mastectomy can be performed safely in patients with at least 4 cm or more of the length of the long axis of the areola. Also, type C is considered far superior as regards cost and cosmetic outcomes, because the patients, who desire to receive postoperative nipple-areolar plasty, are significantly fewer. Since April 2010, we have been trying to apply the nipple-areolar complex made with silicon material (Figure 4) instead of surgical approach. 

Axillary incision is additionally performed in all type B and type C skin incisions at our institution. This is primarily because, in this case series: (1) it was difficult to perform total mastectomy due to the small average areolar diameter (3.7 cm) [19], (2) 65% of the cases underwent complete level I-III ALND, and (3) 97% of the cases chose a microvascularly augmented TRAM flap that required microscopic vascular anastomosis not only in the DIEP flap but also in the TRAM flap, and the plastic surgeon preferred an axillary incision, in order to use thoracodorsal vessels. In fact, an axillary incision is hardly noticeable when seen from the front and we believe it has no influence on the cosmetic outcomes.

Currently in Japan, it is difficult to perform breast reconstruction using implants because of some problems with the medical insurance system—this is the reason why 99% of the cases underwent reconstruction using autogenous tissues. 

Compared with NSSM, it is more difficult to ensure a clear operative field in SSM, and SSM involves more extensive subcutaneous dissection. Therefore, the surgery took longer and the intraoperative blood loss tended to be greater in the first half of the study. In the second half, however, the duration of the SSM procedure and the intraoperative blood loss were comparable to those of NSSM, despite an increase in the percentages of type B and type C incisions, which are supposed to have a narrower field than the type A incision. We believe this finding was greatly influenced by the technical improvement achieved due to the accumulated experience of a single surgeon and due to the bipolar scissors used for subcutaneous dissection in the second half of the study. 

Most of local recurrences after mastectomy occur in the chest wall skin [13, 15]. There was therefore concern that SSM, in which breast skin is conserved to the maximum extent possible, may induce local recurrence. Previous studies have reported that the local recurrence rate is about 2–7% [13, 15–18, 21–23]. It is now widely known that not only the local recurrence rate but also the overall survival in SSM is comparable to those in NSSM, at least for stages 0, I, and II, as seen in the results of our case series. 

A complication common to SSM and NSSM is skin necrosis. Its incidence has been reported to be about 10% [15, 23], and the risk of developing skin necrosis is thought to be equal between the two groups. SSM requires some technical considerations such as (1) avoiding the application of excessive tension to the skin flap or (2) use of a surgical knife to make a thin skin flap just over the tumor. The relationship between skin necrosis observed in SSM and smoking habits has often been examined, and nicotine is thought to be a risk factor for skin necrosis because it reduces capillary blood flow [15–17]. In our case series, skin necrosis occurred in 5% of nonsmokers but in 15% of smokers. 

Although no prospective randomized study that compares SSM and NSSM has been conducted so far, it can be said to be commonly acknowledged that local control, prognosis, and risk of complications are the same for SSM and NSSM, at least in stages 0, I, and II. 

SSM is still considered to be contraindicated for inflammatory breast cancer and breast cancer with skin invasion. Although there have been some studies on the usefulness of SSM in locally advanced breast cancer [24, 25], its application is still controversial. Nonetheless, SSM is considered to be an operative procedure that can be of great benefit to patients with relatively early stage breast cancer who are potential candidates for breast conservation but are ineligible for BCS. 

## 5. Conclusion

When SSM with immediate breast reconstruction is performed in patients with relatively early stage (stages 0–II) breast cancer with tumor size classified as Tis, T1, and T2, the rate of local recurrence, survival, and incidence of postoperative complications are equal to those achieved with NSSM.

Compared with NSSM, SSM is far superior as regards cosmetic outcomes and is expected to remarkably reduce the emotional trauma due to the sense of loss of a breast that is perceived by the patient just after surgery. 

And in SSM, type C is considered far superior as regards cost and cosmetic outcomes, because fewer patients desire to receive postoperative nipple-areolar plasty. 

## Figures and Tables

**Table 1:** Patients and tumor characteristics and stage in SSM and NSSM (%).

	SSM	NSSM
Number of patients	73	129
July 2003–June 2007	*26 (35.6)*	*81 (62.8)*
July 2007–June 2010	*47 (64.4)*	*48 (37.2)*
Age (yrs.)	47.0 ± 9.0	57.7 ± 11.9
Follow-up time (months)	30.0 ± 22.6	41.1 ± 21.3

Microcalcifications on mammography	26 (35.6)	45 (34.9)
Multicentricity	9 (12.3)	15 (11.6)
Nipple discharge	14 (19.2)	13 (10.1)
Distance between nipple and tumor < 20 mm	52 (71.2)	82 (63.6)

Stage 0: TisN0M0	11 (15.1)	10 (7.8)
Stage I:T1N0M0	25 (34.2)	33 (25.6)
Stage IIA	28 (38.4)	58 (43.0)
T1N1M0	*1*	*3*
T2N0M0	*27*	*55*
Stage IIB	9 (12.3)	28 (21.6)
T2N1M0	*8*	*23*
T3N0M0	*1*	*5*

(Neoadjuvant chemotherapy: NAC)	4 (5.5)	5 (3.9)

**Table 2:** Operation characteristics in SSM and NSSM (%).

	SSM	NSSM
Operation (mastectomy) time (min.)	140.1 ± 30.4	130.0 ± 33.8
Blood loss during mastectomy (g.)	212.0 ± 131.8	197.0 ± 146.4

Type of mastectomy		
Bt + ALND	35 (48.0)	98 (75.9)
Bt + SLNB	25 (34.2)	26 (20.2)
Bt + SLNB → ALND	13 (17.8)	5 (3.9)

Bt: total mastectomy, ALND: axillary lymphnode dissection, and SLNB: sentinel lymphnode biopsy.

**Table 3:** Chronological changes in SSM between July 2003–June 2007 and July 2007–June 2010 in SSM (%).

	July 2003–June 2007	July 2007–June 2010
Number of patients	26 (35.6)	47 (64.4)
Operation (mastectomy) time (min.)	148.3 ± 26.9	132.1 ± 50.6
Blood loss during operation (g.)	232.1 ± 174.8	196.6 ± 99.8

Type of skin incision for SSM		
Type A	22 (84.6)	4 (8.5)
Type B	4(15.4)	25 (53.2)
Type C	0 (0)	18 (38.3)

Type of reconstruction following SSM		
LDMC flap	4 (15.4)	6 (12.8)
TRAM flap	18 (69.2)	15 (31.9)
DIEP flap	4 (15.4)	25 (53.2)
Silicon implant	0	1 (2.1)

LDMC: latissimus dorsi musculocutaneous, TRAM: transverse rectus abdominis musculocutaneous, and DIEP: deep inferior epigastric perforator.

**Table 4:** Relations between nipple-areolar complex and type of incision (%).

	Desire for NAP			No desire for
				NAP
		Received	Not received	
Types A and B	48 (88.9)	*33 (61.1)*	*15 (27.8)*	6 (11.1)
Type C	3 (16.7)	*3 (16.7)*	*—*	15 (83.3)

NAP: nipple-areolar plasty.

**Table 5:** Complications in SSM and NSSM (%).

	SSM	NSSM
Postoperative hemorrhage	1 (1.4)	0
Skin necrosis	3 (4.1)	4 (3.1)
DVT	1 (1.4)	0
Flap loss	1 (1.4)	0
Fat lysis of flap with infection	2 (2.7)	0
Hernia at donor site	2 (2.7)	0

DVT: deep vein thrombosis.

**Table 6:** Relation between smoking and complications in SSM(%).

	Smoker	Nonsmoker
Number of patients	20	53
Troubles of skin and flap	3 (15.0)	3 (5.7)
DVT	1 (5.0)	0

DVT: deep vein thrombosis.
